# RNA–Mediated Epigenetic Heredity Requires the Cytosine Methyltransferase Dnmt2

**DOI:** 10.1371/journal.pgen.1003498

**Published:** 2013-05-23

**Authors:** Jafar Kiani, Valérie Grandjean, Reinhard Liebers, Francesca Tuorto, Hossein Ghanbarian, Frank Lyko, François Cuzin, Minoo Rassoulzadegan

**Affiliations:** 1 University of Nice Sophia Antipolis, UFR Sciences, Nice, France; 2 Inserm UMR1091, CNRS UMR7277, Nice, France; 3 Division of Epigenetics, DKFZ-ZMBH Alliance, German Cancer Research Center, Heidelberg, Germany; Medical Research Council Human Genetics Unit, United Kingdom

## Abstract

RNA–mediated transmission of phenotypes is an important way to explain non-Mendelian heredity. We have previously shown that small non-coding RNAs can induce hereditary epigenetic variations in mice and act as the transgenerational signalling molecules. Two prominent examples for these paramutations include the epigenetic modulation of the Kit gene, resulting in altered fur coloration, and the modulation of the Sox9 gene, resulting in an overgrowth phenotype. We now report that expression of the Dnmt2 RNA methyltransferase is required for the establishment and hereditary maintenance of both paramutations. Our data show that the Kit paramutant phenotype was not transmitted to the progeny of *Dnmt2^−/−^* mice and that the Sox9 paramutation was also not established in *Dnmt2^−/−^* embryos. Similarly, RNA from Dnmt2-negative *Kit* heterozygotes did not induce the paramutant phenotype when microinjected into Dnmt2-deficient fertilized eggs and microinjection of the miR-124 microRNA failed to induce the characteristic giant phenotype. In agreement with an RNA–mediated mechanism of inheritance, no change was observed in the DNA methylation profiles of the *Kit* locus between the wild-type and paramutant mice. RNA bisulfite sequencing confirmed Dnmt2-dependent tRNA methylation in mouse sperm and also indicated Dnmt2-dependent cytosine methylation in *Kit* RNA in paramutant embryos. Together, these findings uncover a novel function of Dnmt2 in RNA–mediated epigenetic heredity.

## Author Summary

The possibility of a mode of inheritance distinct from the Mendelian model has been considered since the early days of genetics. Only recently, however, suitable experimental models were created. We now see the development of new experimental systems detecting non-Mendelian inheritance in a variety of organisms, from worms to mice. We have previously shown that RNA molecules act as transgenerational inducers of epigenetic variations in mice. We are currently using Mendelian genetics to dissect the factors involved in RNA–mediated transgenerational signalling. By showing an absolute requirement for Dnmt2 in this process, our study extends our knowledge of this still somewhat enigmatic protein. We confirmed that RNA rather than DNA methylation by the protein is involved in epigenetic heredity, and our genetic results indicate a requirement during an early step in the reproductive process, between parental gametogenesis and the preimplantation stage.

## Introduction

Experimental results on model animals ranging from *Caenorhabditis* and *Drosophila* to the mouse have recently provided support for a mode of epigenetic heredity distinct from the canonical Mendelian rules [1]–[10]. These findings may help in understanding unexpected epidemiological results showing paternal transmission of pathological states over several generations [11]–[13] and provide at least partial solutions to the ‘missing heritability’ problem raised by genomic analyses [11], [14]. Several of the current experimental systems point to RNA as the transgenerational signalling molecule, sperm RNA [15] in the case of paternal heredity.

One important example of RNA-mediated inheritance is provided by the mouse paramutation, where transcriptional activation of a locus is mediated by small non-coding RNAs (sncRNAs). These epigenetic variations were first detected by the hereditary maintenance of the white-tail phenotype of the *Kit* mutation in *Kit^+/+^* offspring of heterozygotes carrying an inactivated allele (*Kit^tmlAlf1/+^*), which was associated with an accumulation of aberrant *Kit* transcripts in germ cells [8]. These RNAs were thought to play a role in the transgenerational transfer of the phenotype, a conclusion strengthened by microinjection assays in naive fertilized eggs. More specifically, oligoribonucleotides with sequences of the transcripts and Kit-specific microRNAs generated the hereditary modification. Similarly, microinjection in eggs of microRNA miR-1 resulted in overexpression of its target *Cdk9* and that of miR-124 in increased expression of *Sox9* during the preimplantation period. The miR-1/Cdk9 paramutants developed cardiac hypertrophy [4] and the miR-124/Sox9 variants a giant phenotype and twin pregnancies [10]. In all three cases, the epigenetic variations were stable over at least three generations of outcrosses and paternal transmission was explained by the transfer of sequence-related molecules in the spermatozoal RNA fraction.

A search for genes involved in paramutation led us to consider a role of the Dnmt2 methyltransferase in RNA mediated epigenetic inheritance. In contrast to other members of the Dnmt family, the Dnmt2 protein catalyses cytosine methylation in RNA substrates, an activity which was at first enigmatic, homozygous null mutants of *Drosophila*, *Arabidopsis* and mouse being viable and fertile under laboratory conditions [16]. Methylation by Dnmt2 was reported to protect tRNAs from cleavage under stress conditions [17] and to be involved in upholding steady state levels of tRNAs [18]. We now report that a homozygous loss-of-function mutation of the *Dnmt2* locus prevents the appearance of epigenetic variants of the *Kit* and *Sox9* loci. Our results indicate that the methyltransferase is not required for expression of the variant phenotype during development. Our data further indicate a Dnmt2-dependent initiation step and suggest a role for Dnmt2 in the homeostasis of sncRNAs in the early embryo.

## Results

### Inheritance of epigenetic variations requires Dnmt2

**Figure 1:**
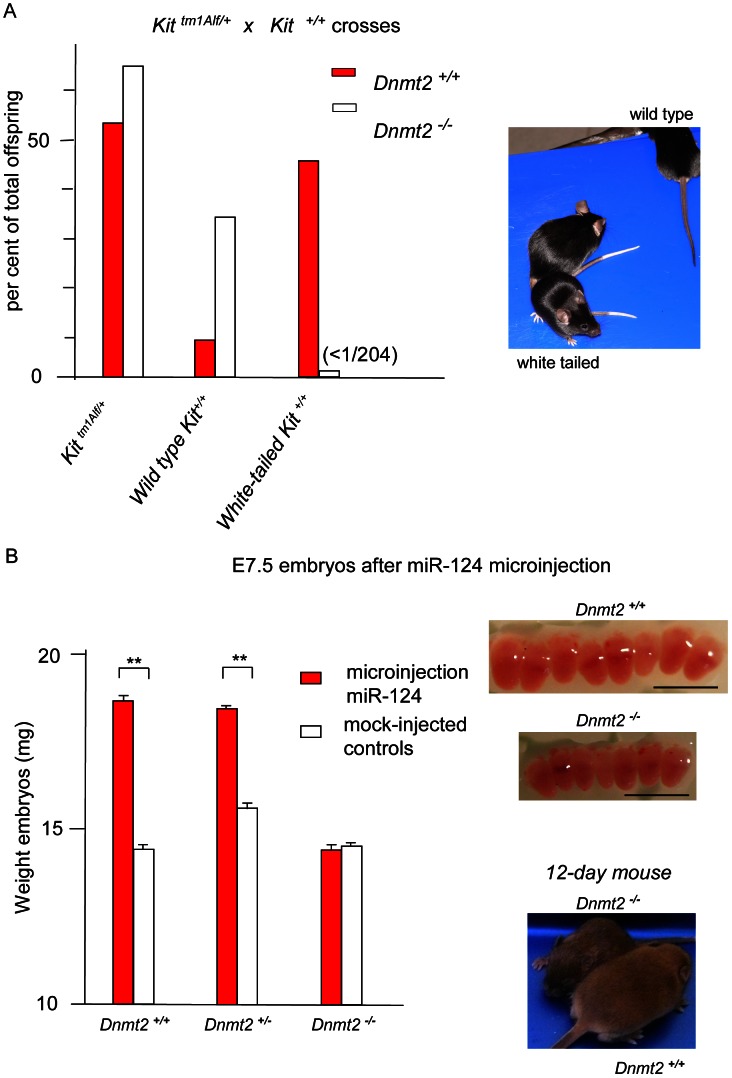
The Kit and Sox9 variant phenotypes are not generated in Dnmt2-negative embryos. A. Kit paramutants in heterozygote mating. In the progeny of crosses between *Kit^tmlAlf1/+^* heterozygotes (either males or females) and *Kit^+/+^* partners, a majority of the *Kit^+/+^* offspring (red boxes) maintained the white-tail phenotype of the mutant shown in the insert photograph. In crosses performed in parallel between isogenic *Dnmt2^−/−^* parents, all the *Kit^+/+^* progeny exhibited the full-color tail phenotype (open boxes). Numeric values and results of *Kit^tmlAlf1/+^* intercrosses are shown in Table 1. B. The Sox9 paramutation induced by microinjection of miR-124 RNA. Fertilized B6D2 eggs were collected following crosses either between *Dnmt2^+/+^* parents, or between a *Dnmt2^+/+^* female and a *Dnmt2^−/−^* male, or between two *Dnmt2^−/−^* parents. Microinjection of single-stranded miR-124 RNA was performed as previously described [4]. E7.5 embryos were collected. In the wild-type and heterozygote *Dnmt2* genotypes, but not in the negative homozygote, the characteristic “giant” phenotype was identified based on the increased size (insert, scaling bar 1 mm) and weight of the embryos. Bars represent the average weight and standard error of the mean (SEM) values for each series of 6 embryos. To minimize variations between foster mothers, controls (microinjected with unrelated RNAs) and miR-124-treated embryos were in each series separately implanted in the two uterine horns of the same mothers. *p*<0.05 for *Dnmt2* negative *versus* wild-type and heterozygote embryos.

The white tail and feet of the *Kit^tmlAlf/+^* heterozygotes (Figure 1A) are immediately recognizable, thus allowing for quantitative studies on relatively large numbers of mice. A non-Mendelian mode of transmission detected in their progenies had initially allowed us to identify a hereditary epigenetic modification of expression of the *Kit^+^* allele (paramutation), which is determined by cognate sncRNAs [8]. We then initiated a search for genes that would affect the establishment and/or maintenance of the paramutated state and considered the Dnmt2 RNA methyltransferase as a possible candidate. We generated 129/Sv mice carrying the heterozygous *Kit* locus and a *Dnmt2* null mutation [16]. The results of crosses between *Kit^tmlAlf1/+^*, *Dnmt2^−/−^* parents are summarized in Figure 1A, with a more detailed presentation in Table 1 and Figure S1. The *Dnmt2^+/+^* control crosses yielded the expected frequency of Kit paramutants (*Kit^+/+^* genotype with the white-spotted phenotype of the mutant). In contrast, in the progeny of two *Dnmt2^−/−^* parents, segregation of the phenotypes strictly corresponded to the *Kit* genotype. Crosses with *Kit^+/+^*, *Dnmt2^+/+^* mice of the wild type, full tail color *Kit^+/+^*, *Dnmt2^−/−^* offspring failed to restore the modified state. A role of the genetic background was excluded because the results were reproduced in C57BL/6 and in B6D2F1 hybrids (Table S1).

**Table 1:** Kit epigenetic variants are not generated in the *Dnmt2^−/−^* genotype.

Exp #		Parents (male×female)	Total progenies (number of litters)	*Kit^tmlAlf1/+^*	*Kit^+/+^* white tail	*Kit^+/+^*
1	*Dnmt2 ^+/+^*	*Kit^tmlAlf1/+^*×*Kit^tmlAlf1/+^*	76 (8)	44	27	5
	*Dnmt2 ^+/+^*	*Kit^+/+^*×*Kit^tmlAlf1/+^*	61 (9)	25	31	5
	*Dnmt2 ^+/+^*	*Kit^tmlAlf1/+^*×*Kit^+/+^*	54 (8)	24	27	3
	*Dnmt2 ^−/−^*	*Kit^tmlAlf1/+^*×*Kit^tmlAlf1/+^*	86 (12)	59	0	27
	*Dnmt2 ^−/−^*	*Kit^+/+^*×*Kit^tmlAlf1/+^*	144 (16)	95	0	49
	*Dnmt2 ^−/−^*	*Kit^tmlAlf1/+^*×*Kit^+/+^*	157 (16)	96	0	61

			Reimplanted embryos (10)	Living births			
2	*Dnmt2 ^−/−^*	*Kit^tmlAlf1/+^*×*Kit^+/+^*	77	75	46	0	29

Progenies of the indicated crosses were individually genotyped by PCR determination of the LacZ marker of the *Kit^tmlAlf1/+^* allele. The epigenetic Kit variants maintain the white-tail phenotype of the mutant with the *Kit^+/+^* homozygote genotype.

In Experiment #3, fertilized eggs were collected at day E 0.5 and implanted into wild type foster mothers. The fact that nearly all fertilized eggs (75 out of 77) developed into healthy progenies excludes any selective developmental arrest during development.

The regular segregation of the Kit^+^ phenotype in *Dnmt2^−/−^* crosses could have been explained by the selective mortality of variant embryos during development. However, further analysis argued against this possibility. As shown in Table 1 (Exp #2), all the embryos generated in 10 crosses between *Kit^tmlAlf1/+^ Dnmt2^−/−^* males and *Kit^+/+^ Dnmt2^−/−^* females were transplanted at the one-cell stage into *Dnmt2^+/+^* foster mothers and 75 living births were obtained from 77 transplants. None of the *Kit^+/+^* progenies showed the variant phenotype under these conditions, thus excluding embryonic lethality.

### The parental methyltransferase is sufficient for transgenerational epigenetic variation

Genetic analysis identified an initial period of establishment of the epigenetic variation. In crosses between *Kit^tmlAlf1/+^*, *Dnmt2^+/−^* males and either *Dnmt2^−/−^* or *Dnmt2^+/−^* females, a fraction of the *Kit^+/+^ Dnmt2^−/−^* offspring showed the white-spotted phenotype (Table 2). This Dnmt2-negative Kit paramutant progeny was generated with a frequency identical to that in *Dnmt2^+/+^* crosses. However, when these mice were subsequently crossed to wild-type partners, they did not further transmit the white tail phenotype. In other words, the epigenetic state was initially maintained in the *Dnmt2^−/−^* genotype during somatic development but was heritable only from a parent with an intact *Dnmt2* allele. We conclude that Dnmt2 activity is critical during parental gametogenesis and/or in fertilized eggs. The resulting change in *Kit* expression in early stem cells can then be maintained in melanoblast stem cells in a Dnmt2-independent manner and results in the defect in their migration during early development responsible for the pigmentation of the adult tail.

**Table 2:** The epigenetic Kit modification is maintained in the *Dnmt2 ^−/−^* progeny of heterozygote parents but not further transmitted.

Cross #	Parents (male×female)	Total births	*Dnmt2 ^−/−^*	*Kit^tmlAlf1/+^*	*Kit^+/+^* white tail	*Kit^+/+^*
1	*Kit^tmlAlf1/+^ Dnmt2 ^+/−^*×*Kit^tmlAlf1/+^ Dnmt2 ^+/−^*	23	6	2	4	0
2	*Kit^tmlAlf1/+^ Dnmt2 ^+/−^*×*Kit^tmlAlf1/+^ Dnmt2 ^−/−^*	25	14	9	2	3
3	*Dnmt2 ^−/−^* Kit^*^×*Dnmt2 ^+/+^ Kit^+/+^*	25	0	0	0	25

Analysis of 3 litters for each cross. In crosses #1 and 2, *Kit* genotypes are indicated in the *Dnmt2 ^−/−^* fraction of the progeny. In cross #3, the male parent was a *Dnmt2 ^−/−^* homozygote with a modified Kit allele born from cross #2. When mated with a wild type female, only non-modified *Kit^+^* alleles were transmitted to the progeny.

Dnmt2 is known to be expressed in oocytes and preimplantation embryos [19] and we detected both Dnmt2 RNA and protein in fractionated male germ cells in spermatocytes, round and elongated spermatids (Figure S3). We also analysed the methylation patterns in mouse sperm of the C38 target site in two established Dnmt2 substrates. The results showed high levels of C38 methylation for tRNA(Asp) and tRNA(Gly) in sperm from wild type mice (Figure S4). This methylation was substantially reduced in sperm from *Dnmt2^−/−^* mice (Figure S4), which provided confirmation for the enzymatic activity of Dnmt2 in the male germline.

### RNA microinjection into *Dnmt2^−/−^* fertilized eggs

Further support for a role of Dnmt2 in the inheritance of epigenetic variation was obtained from RNA microinjection experiments. We had previously shown that microinjection into naive fertilized eggs of either RNA extracted from *Kit^tmlAlf1/+^* tissues, or the cognate microRNAs, or oligoribonucleotides with transcript sequences induced the heritable phenotype modification. We then used these assays to compare the efficiency of RNA preparations from *Dnmt2^−/−^* and *Dnmt2^+/+^ Kit^tmlAlf1/+^* heterozygotes. The results showed that RNA from the brains and testis of Dnmt2-deficient *Kit* heterozygotes did not induce the modified phenotype (Table 3). In subsequent experiments, an oligoribonucleotide with a sequence from the *Kit* mRNA (nt 2123–2150, Table S2) also induced the white-spotted phenotype when microinjected into wild-type one-cell embryos (Table 4). We also tested a form of the same Kit oligoribonucleotide in which all cytosines were methylated. This RNA, indicated as ‘Kit2123–2150met’ in Table 4, was more efficient in inducing the modified phenotype in *Dnmt2^+/+^* embryos but inefficient in the Dnmt2-deficient background, indicating a requirement for Dnmt2 expression in the embryo. We conclude that, while methylation of the inducer RNA is required for optimal efficiency, the methyltransferase is still needed in the most early embryonic period.

**Table 3:** RNA of *Dnmt2 ^−/−^ Kit^tmlAlf1/+^* heterozygotes does not induce paramutation in the wild-type one-cell embryo.

*Kit^tmlAlf1/+^*	*Dnmt2* genotype	Injected *Kit^+/+^* embryos	Litters	Total births	White-tailed*
Testis RNA	+/+	44	3	21	10
Brain RNA	+/+	51	4	23	11
Testis RNA	−/−	56	4	27	1
Brain RNA	−/−	59	4	29	0

* *p*<0.05 between RNAs of Dnmt2-positive and negative animals.

**Table 4:** Induction of paramutation by a fragment of the *Kit* mRNA sequence is increased after methylation but requires Dnmt2 expression.

Embryo genotype	Oligoribonucleotide	Injected embryos	Total births (litters)	White-tailed mice
*Dnmt2 ^+/+^*	Kit 2123–2150	80	28 (7)	4
*Dnmt2 ^+/+^*	Kit2123–2150met	80	38 (8)	13§
*Dnmt2 ^−/−^*	Kit2123–2150met	100	45 (9)	0
*Dnmt2 ^+/+^*	buffer	39	19 (4)	1
*Dnmt2 ^+/+^*	miR-124	50	28 (3)	1
*Dnmt2 ^+/+^*	miR-29b	40	21 (3)	1

Microinjection assays were performed comparatively on wild type fertilized eggs and eggs recovered after mating two *Dnmt2 ^−/−^* parents. Controls received either buffer only. Sequence of the Kit oligoribonucleotide is shown in Table S2. MetKit2 is identical to Kit2123–2150 but with all 8 cytosines methylated.

§ *p*<0.05 between the methylated and the non methylated RNAs.

### Dnmt2 requirement in the Sox9/miR-124 paramutation

To extend our analysis to a second example of a mouse paramutation, we tested whether the lack of Dnmt2 would affect the epigenetic modulation of *Sox9* which can be induced by microinjection of the cognate microRNA miR-124 and of *Sox9* transcript fragments in *Dnmt2^+/+^* embryos [4]. The miR-124/Sox9 variants were characterized by augmented numbers of blastocyst stem cells and, as a result, overgrowth of the embryo and adult body and frequent twin pregnancies. Following microinjection of miR-124 into *Dnmt2^−/−^* fertilized eggs, E7.5 embryos were identical to controls (Figure 1B) and not oversized as the *Dnmt2^+/+^* Sox9 paramutants. We concluded that the paramutation of *Sox9* is also dependent on Dnmt2 expression.

### Unchanged DNA methylation and hydroxymethylation profiles at the *Kit* locus

**Figure 2:**
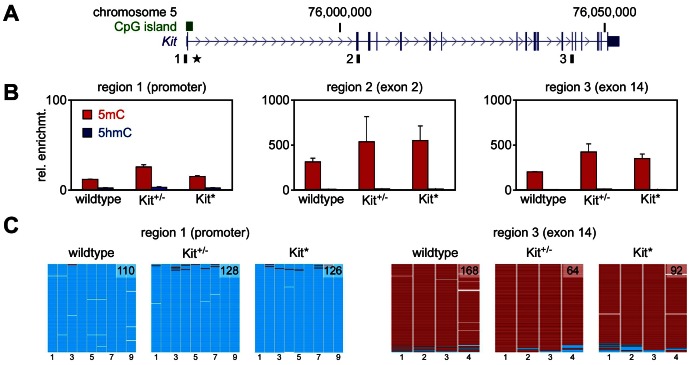
DNA methylation analysis. A. Structure of the *Kit* transcription unit on mouse chromosome 5. Regions analyzed by (h)meDIP are indicated as black boxes, the promoter-associated CpG island is shown as a green box and the transgene insertion site of the *Kit^tmlAlf1/+^* allele is marked by an asterisk. B. (h)meDIP analysis of genomic DNA from mouse testes. Immunoprecipitated DNA was amplified by locus-specific qPCR and enrichments were calculated relative to the unmethylated actin control. 5mC values are shown as red bars and 5hmC values as blue bars. Diagrams show the results of at least three independent experiments, standard errors of the mean are indicated by error bars. C. Bisulfite sequencing analysis of genomic DNA from testes. Methylation maps show 454 sequencing reads (rows) and the methylation status of 9 CpGs (columns) within the Kit promoter and 4 CpGs from the Kit exon 14 region. Methylated CpGs are shown in red, unmethylated CpGs in cyan and gaps in white. Numbers in methylation maps indicate the number of sequencing reads.

Modified patterns of DNA methylation have been reported in various instances of epigenetic variation [ref. 20 for review] including the maize paramutation [21]. We used methylated DNA immunoprecipitation (meDIP) to determine the DNA methylation status of the Kit locus in testis DNA from wild type, *Kit^tmlAlf1/+^* and *Kit^+/+^* paramutant mice. Assays were developed for three distinct regions covering the *Kit* promoter, exon 2 and exon 14, respectively (Figure 2A). The results indicated only background levels of methylation in the promoter, and substantial methylation in the two intragenic regions (Figure 2B). This pattern was observed for all three genotypes (Figure 2B), indicating that the Kit paramutation is not associated with altered DNA methylation profiles of the locus – although we cannot exclude a localized change in an unknown element at a distance, as described for the *b1* paramutation of maize [21]. In parallel experiments, we also used this approach to determine the DNA hydroxymethylation status of the locus and found that hydroxymethylation levels were invariably low in all genotypes and regions tested (Figure 2B). The meDIP findings were subsequently validated by DNA bisulfite sequencing of testis DNA. The results demonstrated that the *Kit* promoter was completely unmethylated and that the exon 14 region was completely methylated (Figure 2C). This pattern was again observed for all three genotypes (Figure 2C), which further suggests that paramutation is not associated with an altered DNA methylation profile of the *Kit* locus.

### Cytosine methylation analysis of *Kit* RNA molecules

**Figure 3:**
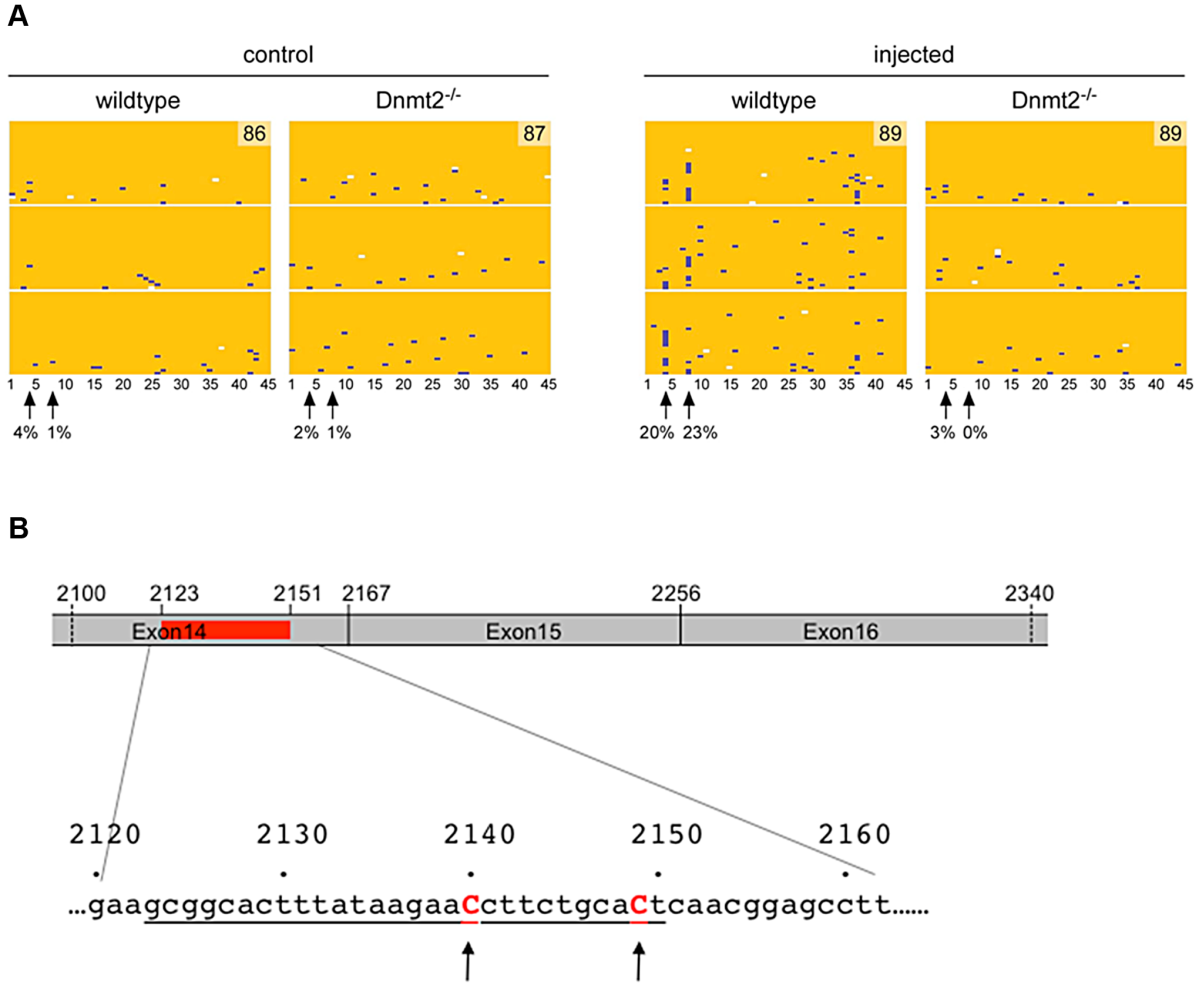
RNA methylation analysis. A. Single-cell embryos were collected after mating of either two wild-type or two *Dnmt2^−/−^* B6/D2 parents. After microinjection of the Kit2123–2150 oligoribonucleotide (representing 28 nt of the mRNA sequence) or buffer, the embryos were transferred to foster mothers (2 for each condition and 10 embryos per foster). At embryonic day E9.5, 6 to 8 embryos were obtained from each foster. Total RNA was prepared separately from each embryo and processed for *Kit* RNA methylation analysis. RNA bisulfite sequencing maps are shown for 45 cytosine residues from the Kit exon 14 region in microinjected wild-type and Dnmt2^−/−^ embryos. Each row represents one sequence read, each column a cytosine residue. Yellow boxes represent unmethylated cytosine residues, blue boxes indicate methylated cytosine residues, sequencing gaps are shown in white. Results are shown for three independent biological replicates, numbers in methylation maps indicate the total number of sequencing reads. Arrows mark two putative cytosine methylation sites, numbers below arrows indicate the site-specific methylation levels. B. Schematic drawing of the sequenced region of *Kit* mRNA. Position of the microinjected oligoribo-nucleotide is shown in red and underlined in the nucleotides sequence.

We also used RNA bisulfite sequencing to analyze the possibility that Dnmt2 might methylate *Kit* transcripts. To this end, we induced the Kit paramutation by microinjection of an oligoribonucleotide (Kit2123–2150) into fertilized eggs obtained from either two *Dnmt2^−/−^* or two wild-type parents. In parallel, we also prepared control embryos that were injected with buffer. RNA was prepared from E9.5 embryos and methylation analysis was performed on the 45 cytosines of a region amplified from *Kit* RNA (nt 2100–2336) that covers the inducer oligoribonucleotide (nt 2123–2150) and overlaps the exon 14∶15∶16 junctions. The results revealed two closely associated cytosines (cytosines #4 and #8, Figure 3) that remained unconverted in a higher fraction of reads, specifically in the microinjected *Dnmt2^+/+^* embryos. Methylation of mRNA by Dnmt2 has not been reported so far and it is possible that our results have been influenced by deamination artifacts. However, the same two methylation sites were identified in three independent biological replicates and were not observed in the control embryos or in the oligoribonucleotide-treated *Dnmt2^−/−^* embryos (Figure 3), which suggests that they might represent genuine methylation marks. Contamination by DNA was excluded by the spliced structure of the sequence. Furthermore, we also tested the methylation pattern of the corresponding genomic sequence. The results showed methylated CpG sites that were clearly distinct from the sites detected in RNA and that were not dependent on the Dnmt2 genotype (Figure S5).

## Discussion

The physiological role of the RNA methyltransferase activity of Dnmt2 has been enigmatic for a long period of time. Dnmt2-mediated tRNA methylation has recently been linked to tRNA stability [17], [18]. However, the widespread occurrence of 5-methylcytosine in RNA [22] may reflect a variety of functions, most of which still remain to be identified. The recurrent general considerations on a regulatory role of noncoding RNAs [reviewed in refs 23], [24] led us to consider a possible physiological function of the methyltransferase in epigenetic regulation. The three instances of RNA-mediated hereditary variation that we reported as paramutations provided suitable experimental models.

We now report that Dnmt2 is required for establishment and hereditary transmission of the epigenetic variation at the *Kit* and *Sox9* loci. This was first revealed for the visible color phenotype of the *Kit* variants, a most classical approach in genetics. It was further confirmed and extended to *Sox9* by microinjection experiments. Our data show that the parental RNAs and synthetic oligoribonucleotide inducers of the epigenetic variations were inefficient in Dnmt2-negative embryos. Evidence for RNA methylation in the inducer oligonucleotide sequence was observed in embryos undergoing the Kit paramutation. Furthermore, while the modified Kit phenotype was never observed in *Dnmt2 ^−/−^* homozygotes born from two parents with the same genotype, it was, however, expressed by genetically identical homozygotes when at least one of their parents was a Dnmt2-positive heterozygote (Table 2). We concluded that the protein is required only during the parental gametogenesis or in the early embryo and not at later developmental stages – except for subsequent transgenerational transfer.

At least two general explanatory models can be considered for the absolute requirement in Dnmt2 in the establishment of the epigenetic change. One model would be based on the knowledge that tRNAs are bona fide substrates of Dnmt2, and that tRNA fragments are highly abundant in mouse sperm [25]. Our data show that at least two tRNAs are methylated in mouse sperm in a Dnmt2-dependent manner (Figure S4), which raises the possibility that methylation-dependent processing of tRNAs [17] could result in the generation of paramutation-inducing sncRNAs. However, we have so far been unable to detect any recognizable phenotypes after microinjection of various tRNAs and tRNA fragments (data not shown). A second model would consider that the inducer small RNAs are maintained only in the *Dnmt2^+/+^* genotype, possibly because they are methylated or complexed with methylated tRNAs. Such a model would also account for the increased efficiency of the methylated synthetic oligoribonucleotides (Table 4). Current preliminary results suggest that exogenous small RNAs introduced in the early embryo are stably maintained only in Dnmt2-positive embryos, leading us to the hypothesis of a protection against endonucleolytic cleavage by methylation in a manner analogous to tRNAs [17]. A control of the maintenance of parental small RNAs at the maternal-zygotic transition would be reminiscent of the mechanisms that, at the same developmental stages, eliminate part of the parental mRNAs [26]. In such a model, the new individual would actively constitute its own set of functional RNAs, both large and small, from the parental stocks.

## Materials and Methods

### Mice and genotyping

The experiments here described were carried out in compliance with the relevant institutional and French animal welfare laws, guidelines and policies. They have been approved by the French ethics committee (Comité Institutionnel d'Ethique Pour l'Animal de Laboratoire; number NCE/2012-54). *Kit^tmlAlf1/+^* heterozygotes were maintained in parallel in the original 129/Sv genetic background and in C57BL/6×DBA/2 F1 hybrids (B6D2). The *Dnmt2^−/−^* homozygote [16] was kindly provided by T. Bestor. Originally maintained on a mixed genetic background, the mutation was backcrossed onto 129/Sv, C57BL/6 and B6D2 genetic backgrounds, in each case for more than ten generations. Genotypes were determined by PCR analysis of *Neo* and *LacZ* expression and by Southern blot hybridization using a genomic probe.

### RNA microinjection

Total brain and testis RNA and oligoribonucleotides with Kit and miRNA sequences were adjusted to a concentration of 1 µg/ml and microinjected into B6D2 fertilized eggs according to established methods of transgenesis [27]. Quality of RNA preparations from the mouse organs was checked by spectroscopic analysis using the Bioanalyzer 2100 apparatus (Agilent Technologies, Santa Clara, CA) (Figure S2). Oligoribonucleotides were obtained from Sigma-Prolabo (sequences provided in Table S2).

### Northern blot

Northern blot analysis was performed by standard methods [28]. For analysis, RNA was extracted with Trizol Reagent (Invitrogen).

### Western blot analysis

Protein extracts for Dnmt2 Western blot were prepared from snap-frozen enriched germ cell populations obtained by homogenization in RIPA Buffer. Testicular fractions were purified by elutriation as described [29]. 20 µg of protein was fractionated onto a 15% denaturing SDS-polyacrylamide gel and transferred to nitrocellulose. The following antibodies were used for immunodetection: rabbit anti-Dnmt2 antibody (Santa-Cruz, Rabbit polyclonal IgH sc-20702, lot: B1903) 1∶100 and rabbit anti-ß-actin antibody (Santa-Cruz, sc-47778, lot: D0907) 1: 250 with peroxidase-coupled goat anti-rabbit secondary antibody (Santa Cruz Biotechnology) 1∶10,000.

### DNA methylation analysis

Methylated DNA immunoprecipitation was performed as described previously [30]. Sequences of PCR primers are shown in Table S3. DNA bisulfite sequencing analysis was performed by using the EpiTect Bisulfite Kit (Qiagen), in combination with 454 sequencing technology (Roche). Sequences of 454 bisulfite sequencing primers are shown in Table S3 and S4. Methylation maps were generated by BISMA [31].

### RNA methylation analysis

Analysis of cytosine methylation in Kit RNA was performed as described [32], with minor modifications. RNA isolated using TRIzol (Invitrogen) was digested with DNase (Promega). An aliquot of 6 µg of RNA dissolved in 20 µl of RNase-free water was mixed with 42.5 µl of “Bisulfite Mix” and 17.5 µl of “DNA Protect” buffer. The RNA was denatured at 70°C for 5 min, followed by 1 h incubation at 60°C. This cycle was repeated 5 times. RNA was isolated from the bisulfite reaction mix using the RNeasy Purification Kit (Qiagen) and treated with 0.5 M Tris-HCl, pH 9 at 37°C for 1 h. Finally, RNA was precipitated and further processed for sequencing, as described previously [32]. This included random barcoding during the reverse transcription reaction to confirm that the sequenced DNA molecules represented different RNA molecules. Sequences of PCR primers are shown in Table S3 and S4. Sperm RNA was prepared as described [10] and analyzed as described previously [18].

### Statistics

Data are expressed as means ± s.e.m. A p-value of less than 0.05 was considered statistically significant.

## Supporting Information

Figure S1 Generation of *Dnmt2^−/−^ Kit* heterozygotes and crosses with *Kit^+/+^* partners. A. The phenotypes of the genetically *Kit^+/+^* offsprings are indicated by colors, green for the wild type (full tail color) and red for the paramutants (white tail). B. Crosses between *Dnmt2^+/−^* parents generate a non heritable, paramutant, phenotype with *Dnmt2^−/−^* genotype. Number of crosses and mice analyzed in a representative series are shown in Table 1.
(PDF)

Figure S2 Gel and electropherogram profiles of mouse total RNA samples using Bioanalyzer 2100. Total RNA from brain and testes of different genotypes was isolated using Trizol (Invitrogen) and RNA was loaded in the 2100 RNA Bioanalyzer (Agilent, Santa Clara, CA). Lane L: size markers. Sharp bands of 28S and 18S ribosomal RNA are quality control of isolated total RNA.
(PDF)

Figure S3 Dnmt2 is expressed up to the late spermatogenic stages. Expression was analyzed by Northern (A) and by Western blotting (B) in testis cells purified by elutriation as described [32].
(PDF)

Figure S4 tRNA methylation heatmaps for tRNA^Asp^ and tRNA^Gly^ in wild-type and Dnmt2^−/−^ sperm. Numbers indicate the number of sequencing reads, arrowheads indicate the Dnmt2 target position (C38).
(JPG)

Figure S5 Cytosine methylation in exon 14 of the *Kit* RNA sequence does not correspond to the sites methylated in the genomic sequence. Bisulfite assays of C-methylation. Empty circles show the position of unmethylated cytosines, filled circles that of methylated cytosines. Top: reverse transcribed-amplified RNA sequences. Bottom: the corresponding sequence in genomic DNA. Each line corresponds to the common pattern of 30 sequences read for each genotype. 1: *Dnmt2 ^+/+^* embryos, 2: *Dnmt2^−/−^* embryos after microinjection of the Kit oligoribonucleotide.
(PDF)

Table S1 Segregation of phenotypes in *Dnmt2^−/−^ Kit* heterozygote crosses in the B6D2 F1 hybrid and C57BL/6 genetic backgrounds.
(DOCX)

Table S2 Oligoribonucleotides for microinjection experiments
(DOCX)

Table S3 Primers for PCR amplification.
(DOCX)

Table S4 Primers for bisulfite sequencing.
(DOCX)
