# Supplementary figures and images for: RNA–Mediated Epigenetic Heredity Requires the Cytosine Methyltransferase Dnmt2
Source: PLoS Genet. 2013 May 23;9(5):e1003498. doi: 10.1371/journal.pgen.1003498 (PMC3662642; doi:10.1371/journal.pgen.1003498)

A

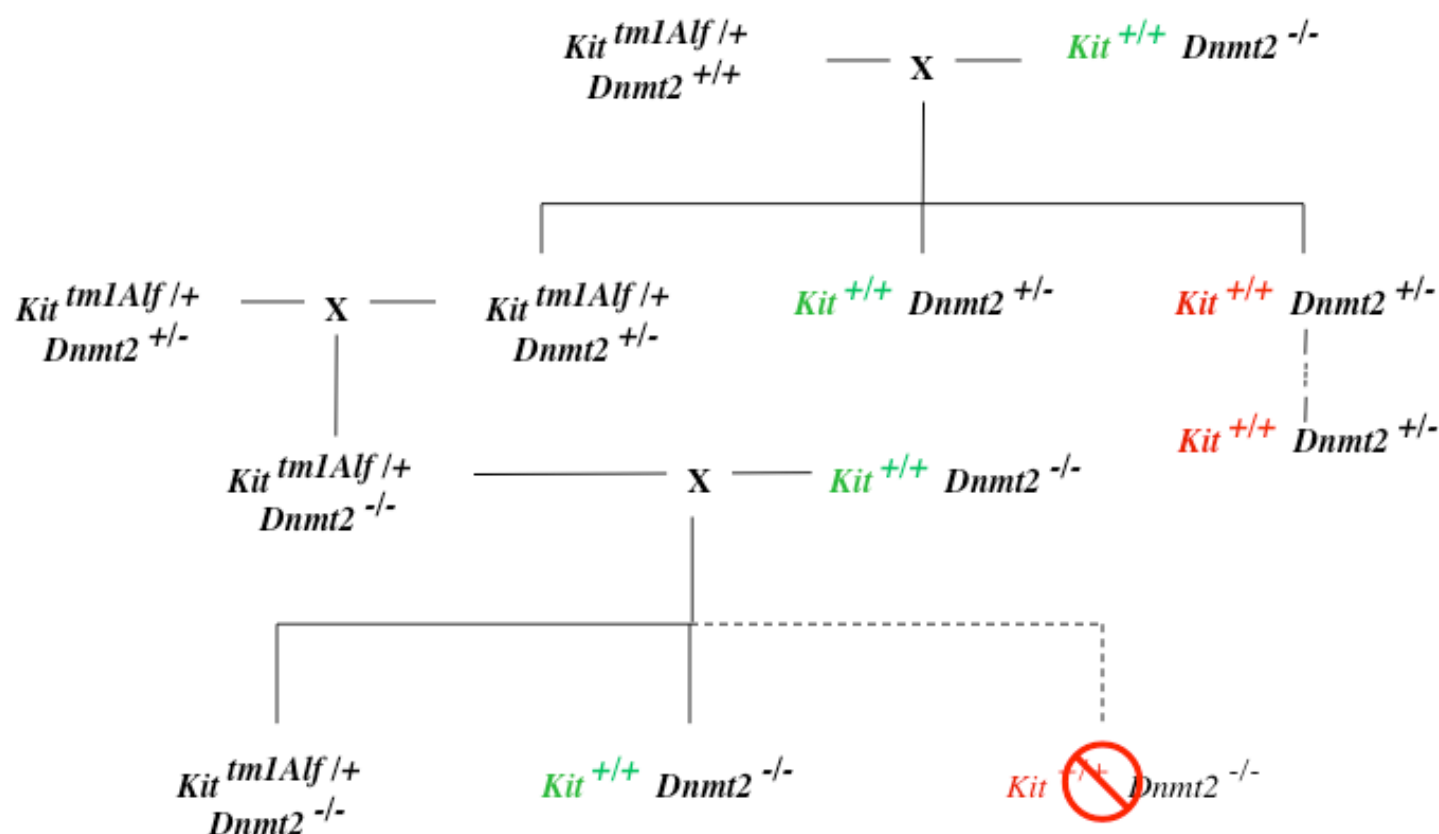

B

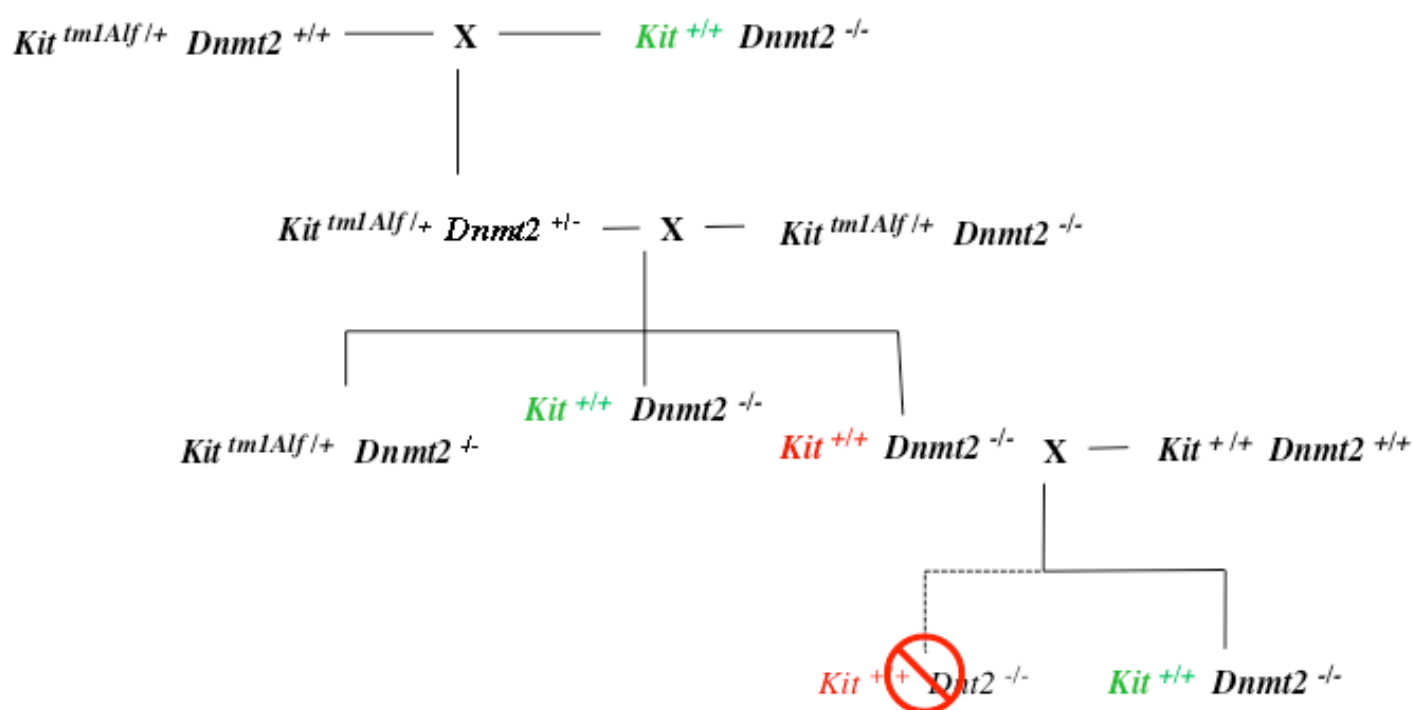

*Kit*<sup>+/+</sup> : full tail color      *Kit*<sup>+/+</sup> : white tail (*Kit*<sup>\*</sup>)

Supplement: Figure S1 — Generation of Dnmt2−/− Kit heterozygotes and crosses with Kit+/+ partners. A. The phenotypes of the genetically Kit+/+ offsprings are indicated by colors, green for the wild type (full tail color) and red for the paramutants (white tail). B. Crosses between Dnmt2+/− parents generate a non heritable, paramutant, phenotype with Dnmt2−/− genotype. Number of crosses and mice analyzed in a representative series are shown in Table 1. (PDF) [file pgen.1003498.s001.pdf]

Kiani et al. Supplemental Figure S2

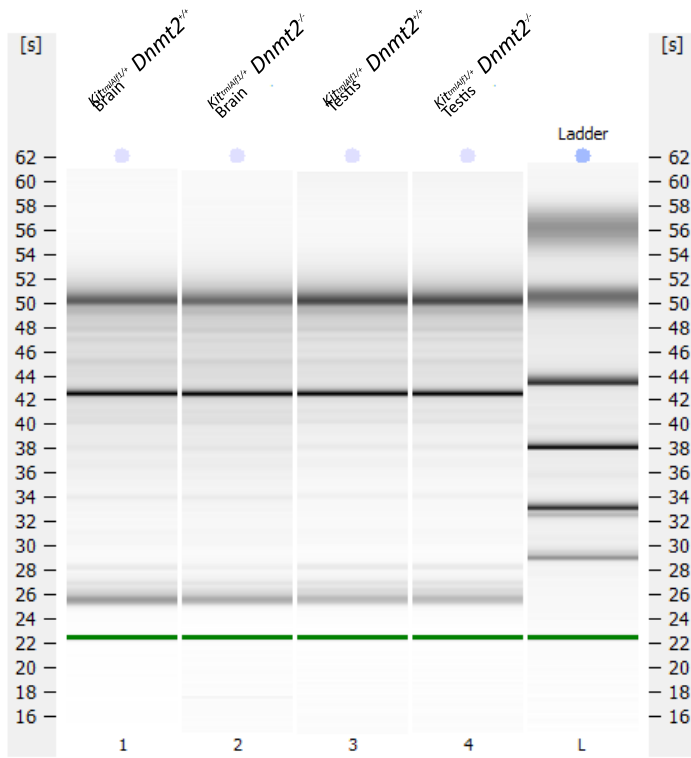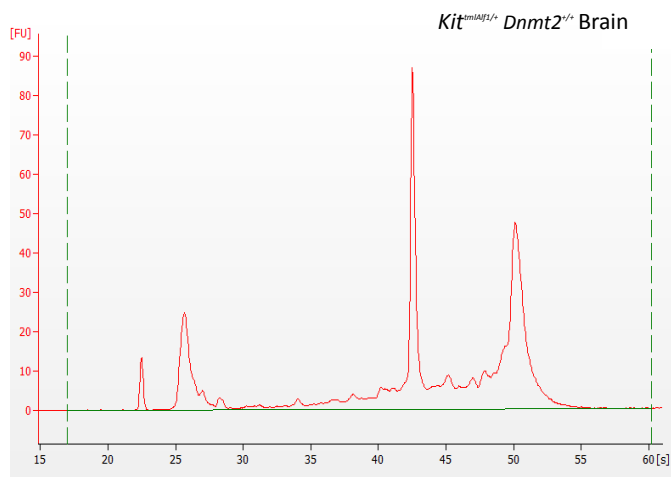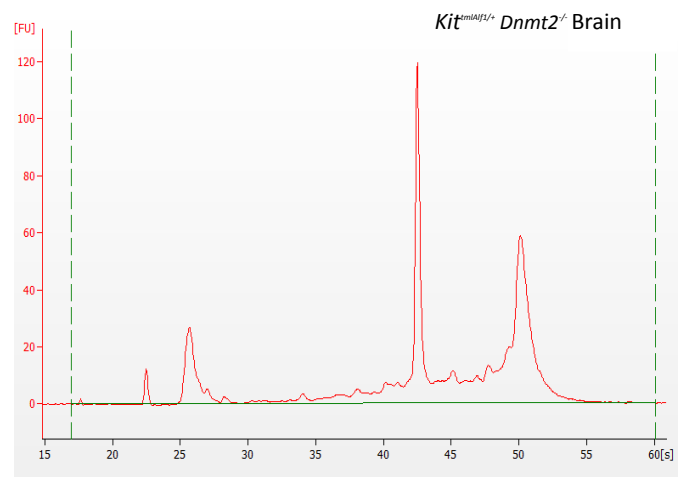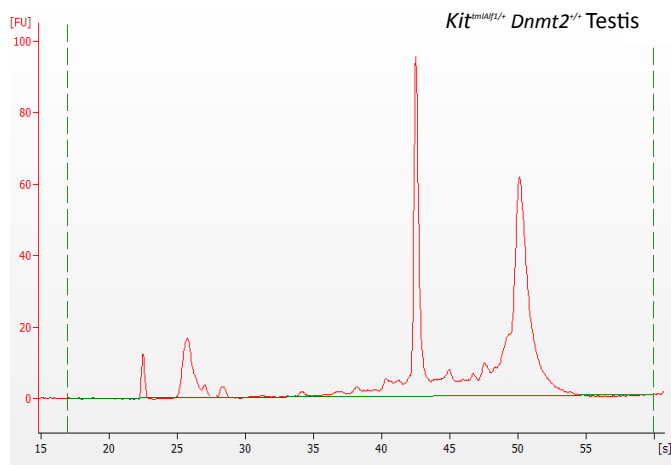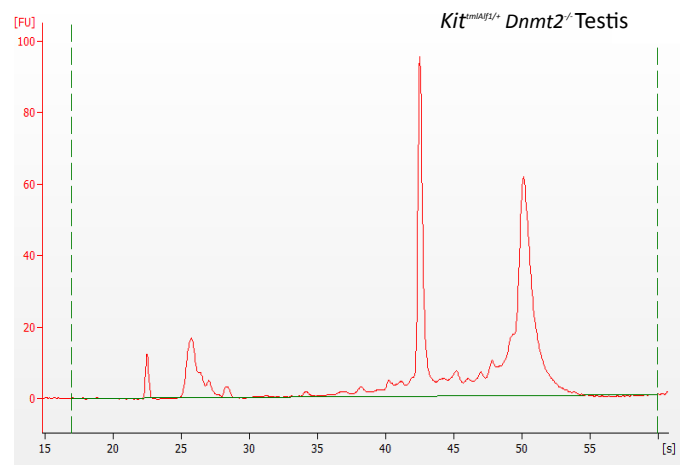

Supplement: Figure S2 — Gel and electropherogram profiles of mouse total RNA samples using Bioanalyzer 2100. Total RNA from brain and testes of different genotypes was isolated using Trizol (Invitrogen) and RNA was loaded in the 2100 RNA Bioanalyzer (Agilent, Santa Clara, CA). Lane L: size markers. Sharp bands of 28S and 18S ribosomal RNA are quality control of isolated total RNA. (PDF) [file pgen.1003498.s002.pdf]

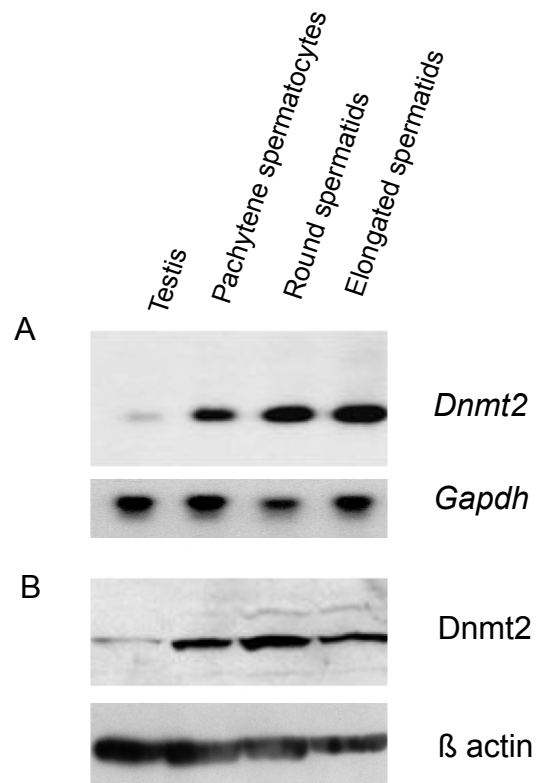

Supplement: Figure S3 — Dnmt2 is expressed up to the late spermatogenic stages. Expression was analyzed by Northern (A) and by Western blotting (B) in testis cells purified by elutriation as described [32]. (PDF) [file pgen.1003498.s003.pdf]

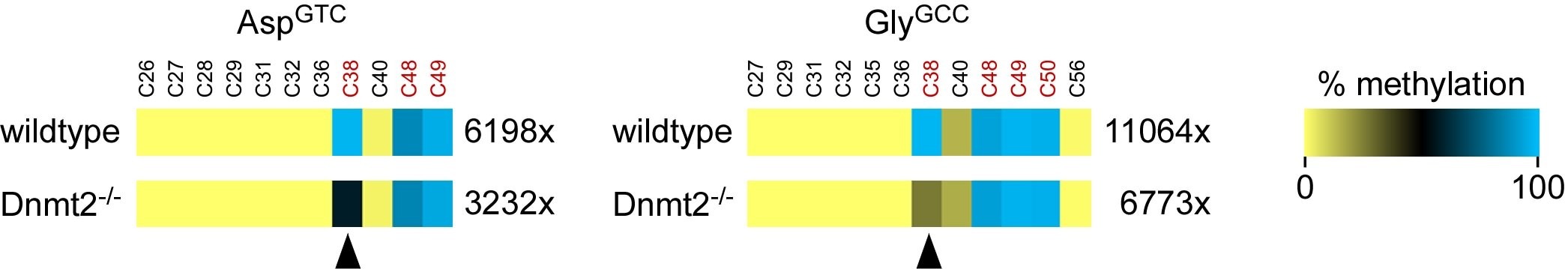

Supplement: Figure S4 — tRNA methylation heatmaps for tRNAAsp and tRNAGly in wild-type and Dnmt2−/− sperm. Numbers indicate the number of sequencing reads, arrowheads indicate the Dnmt2 target position (C38). (JPG) [file pgen.1003498.s004.jpg]

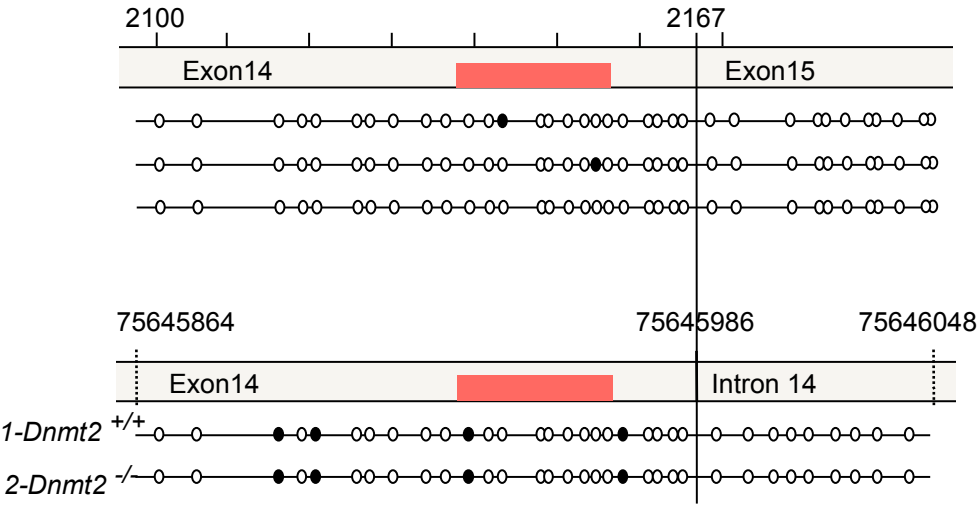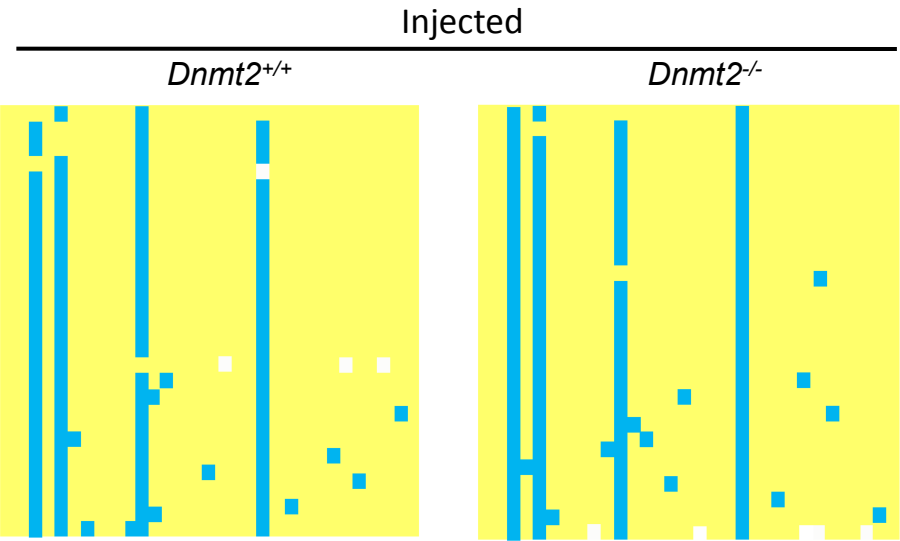

Supplement: Figure S5 — Cytosine methylation in exon 14 of the Kit RNA sequence does not correspond to the sites methylated in the genomic sequence. Bisulfite assays of C-methylation. Empty circles show the position of unmethylated cytosines, filled circles that of methylated cytosines. Top: reverse transcribed-amplified RNA sequences. Bottom: the corresponding sequence in genomic DNA. Each line corresponds to the common pattern of 30 sequences read for each genotype. 1: Dnmt2 +/+ embryos, 2: Dnmt2−/− embryos after microinjection of the Kit oligoribonucleotide. (PDF) [file pgen.1003498.s005.pdf]
