# Supplementary material for: RNA–Mediated Epigenetic Heredity Requires the Cytosine Methyltransferase Dnmt2
Source: PLoS Genet. 2013 May 23;9(5):e1003498. doi: 10.1371/journal.pgen.1003498 (PMC3662642; doi:10.1371/journal.pgen.1003498)
Supplement: Table S1 — Segregation of phenotypes in Dnmt2−/− Kit heterozygote crosses in the B6D2 F1 hybrid and C57BL/6 genetic backgrounds. (DOCX) [file pgen.1003498.s006.docx]

**Supplemental Table S1. The absence of Kit* paramutants (*Kit^+/+^* genotype and mutant phenotype)
is not unique to the 129/Sv genetic background**

| Genetic background $ | Parents  (male x female) | Total progenies  (number of litters) | *Kit^tmlAlf1/+^* | | *Kit^+/+^*  white tail | | *Kit^+/+^* | |  |
| --- | --- | --- | --- | --- | --- | --- | --- | --- | --- |
| B6/D2 *Dnmt2 ^+/+^* | *Kit^tmlAlf1/+^* x *Kit^tmlAlf1/+^* | 4 | | 11 | | 9 | | 4 | |
| B6/D2 *Dnmt2 ^+/+^* | *Kit^+/+^*x *Kit^tmlAlf1/+^* | 3 | | 8 | | 12 | | 3 | |
| B6/D2 *Dnmt2 ^+/+^* | *Kit^tmlAlf1/+^* x *Kit^+/+^* | 2 | | 6 | | 7 | | 2 | |
| B6/D2 *Dnmt2 ^-/-^* | *Kit^tmlAlf1/+^* x *Kit^tmlAlf1/+^* | 5 | | 18 | | 0 | | 11 | |
| B6/D2 *Dnmt2 ^-/-^* | *Kit^+/+^* x *Kit^tmlAlf1/+^* | 3 | | 11 | | 0 | | 7 | |
| B6/D2 *Dnmt2 ^-/-^* | *Kit^tmlAlf1/+^* x *Kit^+/+^* | 4 | | 15 | | 0 | | 10 | |
| C57BL/6 *Dnmt2 ^+/+^* | *Kit^tmlAlf1/+^*x *Kit^tmlAlf1/+^* | 4 | | 11 | | 10 | | 2 | |
| C57BL/6 *Dnmt2 ^+/+^* | *Kit^+/+^* x *Kit^tmlAlf1/+^* | 3 | | 9 | | 10 | | 2 | |
| C57BL/6 *Dnmt2 ^+/+^* | *Kit^tmlAlf1/+^* x *Kit^+/+^* | 2 | | 6 | | 7 | | 1 | |
| C57BL/6 *Dnmt2 ^-/-^* | *Kit^tmlAlf1/+^* x *Kit^tmlAlf1/+^* | 7 | | 34 | | 0 | | 15 | |
| C57BL/6 *Dnmt2 ^-/-^* | *Kit^+/+^* x *Kit^tmlAlf1/+^* | 3 | | 12 | | 0 | | 9 | |
| C57BL/6 *Dnmt2 ^-/-^* | *Kit^tmlAlf1/+^* x *Kit^+/+^* | 2 | | 9 | | 0 | | 5 | |
| The *Dnmt2 ^-^* mutation, initially in a mixed genetic background, was backcrossed for more than 10 generations into the C57BL/6 and B6D2 (C57BL/6 x DBA/2 F1) genetic backgrounds | | | | | | | | | |
