# Supplementary material for: RNA–Mediated Epigenetic Heredity Requires the Cytosine Methyltransferase Dnmt2
Source: PLoS Genet. 2013 May 23;9(5):e1003498. doi: 10.1371/journal.pgen.1003498 (PMC3662642; doi:10.1371/journal.pgen.1003498)
Supplement: Table S2 — Oligoribonucleotides for microinjection experiments (DOCX) [file pgen.1003498.s007.docx]

Supplemental Table S2. Oligoribonucleotides in microinjection experiments

| Kit2123-2150  Kit2123-2150met | GCGGCACUUUAUAAGAACCUUCUGCACU  GmCGGmCAmCUUUAUAAGAAmCmCUUmCUGmCAmCU |  |
| --- | --- | --- |
| mmu-miR-124  mmu-miR-29b | UAAGGCACGCGGUGAAUGCC  UAGCACCAUUUGAAUCAGUGUU | |
| Nucleotide numbers refer to the Kit mRNA sequence (accession number Y00864).  mC: 5-methylcytosine. | | |
