# Supplementary material for: RNA–Mediated Epigenetic Heredity Requires the Cytosine Methyltransferase Dnmt2
Source: PLoS Genet. 2013 May 23;9(5):e1003498. doi: 10.1371/journal.pgen.1003498 (PMC3662642; doi:10.1371/journal.pgen.1003498)
Supplement: Table S3 — Primers for PCR amplification. (DOCX) [file pgen.1003498.s008.docx]

**Supplemental Table S3. Primers for qPCR, PCR, Northern and Genotyping**

| Gene | Primer type | Sequence (5’-3’) |
| --- | --- | --- |
| *Kit* | qPCR Forward | AAGTGGATGGCACCAGAGAG |
|  | qPCR Reverse | GATCAAGGAAGGCTTCCGGA |
|  | meDIP prom Forward | CTGGGAGGAGGGCTGGAG |
|  | meDIP prom Reverse | CTCTCTCTGCTACAGCTCTCGCC |
|  | meDIP ex2 Forward | AGCCACGTCTCAGCCATCTG |
|  | meDIP ex2 Reverse | GTATGTGCCCGTGCGAGTG |
|  | meDIP ex14 Forward | AGCCACTGAATCTGAATGTTAATAGC |
|  | meDIP ex14 Reverse | GGCTCCGTTGAGTGCAGAAG |
|  | BS promoter Forward | TTAGGGAGTATTTGTTAGGTGGTTG |
|  | BS promoter Reverse | CTATACCCTCTAAAACCAAAAACCC |
|  | BS exon 14 Forward | TTTATTTTGGTTATTATAGAATATTGTTGT |
|  | BS exon 14 Reverse | AATCAACCTTACCAAAAAAACTCC |
| *Act* | meDIP actin Forward | AGCCAACTTTACGCCTAGCGT |
|  | meDIP actin Reverse | TCTCAAGATGGACCTAATACGGC |
| *tRNA^Asp^* | BS forward | TGTTAGTATAGTGGTGAGTAT |
|  | BS reverse | CTCCCCATCAAAAAATTA |
| *tRNA^Gly^* | BS Forward | GGTGGTTTAGTGGTAGAATT |
|  | BS Reverse | TACATTAACCAAAAATC |
| *Gapdh* | Northern Forward | CTCTGGAAAGCTGTGGCGTGATGGC |
|  | Northern Reverse | AGTCGCAGGAGACAACCTGGTCCTCA |
| *Dnmt2* | Northern Forward | TTCCTTGAAGATGGCGACACAGATGAGTAC |
|  | Northern Reverse | CAAATTCTGGAGGAAATCCCTGGAGAT |
| *Dnmt2* | Genotyping Forward 1 | CATACAATGCCCGTGTGAGTTCTTAAGG |
|  | Genotyping Reverse 1 | CGTGTGTCTAAATGGCTTGAGTACAGT |
|  | Genotyping Forward 2 | CAGAGTGAGGCTACAGGGCT |
|  | Genotyping Reverse 2 | AGGGGAAAACAGTCAACAGG |
