# Supplementary material for: RNA–Mediated Epigenetic Heredity Requires the Cytosine Methyltransferase Dnmt2
Source: PLoS Genet. 2013 May 23;9(5):e1003498. doi: 10.1371/journal.pgen.1003498 (PMC3662642; doi:10.1371/journal.pgen.1003498)
Supplement: Table S4 — Primers for bisulfite sequencing. (DOCX) [file pgen.1003498.s009.docx]

**Table S4. Primers for bisulfite sequencing of Kit transcripts**

| Gene | Primer type | Sequence (5’-3’) |
| --- | --- | --- |
| Kit | RT primer | CCTTGCCAGCCCGCTCAGNNNNNACTCAACAAATCATCCAAATCCA |
|  | Forward 1 | AAAGTAAGAAGAGTAGGTAGAAG |
|  | Forward 2 | GAATTTTTTGAGAAGGAAG |
|  | Reverse | CTATGCGCCTTGCCAGCCCGCTCAG |
| Kit | Forward | TTGTTATGGTGATTTTTTGAA |
|  | Reverse | AAACCCTTATAACCCCATAAACT |
| N is any one of the four nucleotides, randomly introduced for ‘bar coding’ [24]. | | |
